# Supplementary material for: Choosing what we like vs liking what we choose: How choice-induced preference change might actually be instrumental to decision-making
Source: PLoS One. 2020 May 18;15(5):e0231081. doi: 10.1371/journal.pone.0231081 (PMC7233538; doi:10.1371/journal.pone.0231081)
Supplement: S1 Text — In particular, this note summarizes numerical simulations performed under so-called drift-diffusion decision models. (PDF) [file pone.0231081.s001.pdf]

Choosing what we like vs liking what we choose: supplementary information

This note discusses the relationship between response time (RT) and choice-induced preference change (CIPC), from a computational standpoint.

Recall that we hypothesize that value reassessment during decision-making is a critical determinant of CIPC. This might tempt one to predict that longer RT should lead to greater CIPC. The logic of this idea would be that difficult decisions cause people to deliberate longer, which in turn allows for larger changes in value estimates. Although this reasoning may not hold in general (cf., e.g., if effort intensity varies over trials), we here summarize a computational scenario that would explain why CIPC may increase with RT.

More precisely, we will describe numerical simulations of a drift-diffusion model (DDM). In brief, these models assume that decisions are triggered when the so-called "decision variable" hits a predefined bound. Here, we assume that the decision variable or DV is the difference between option values, which evolves according to the following random walk:

$$DV_{t+1} = DV_t + DR + \varepsilon_t$$

where the drift rate DR captures the rate at which value-relevant information can be assimilated, ε_t is a random perturbation term, and t indexes within-trial decision time. In the following simulations, the initial DV is drawn at random, and a decision is triggered when the DV hits a predefined bound. Note that we assume that the DDM bounds are collapsing with time. This is in fact a property of optimal DDMs for value-based decision making (Tajima et al., 2016).

Of particular interest here are:

- The response time RT, i.e. the time at which the bound is hit.
- The final DV, i.e. the bound's height when it is hit. This is because CIPC can be measured in terms of the difference between the final and the initial DV (see below).

Figure 1 below shows two typical sample paths (i.e. two trials) simulated under the DDM with collapsing bounds:

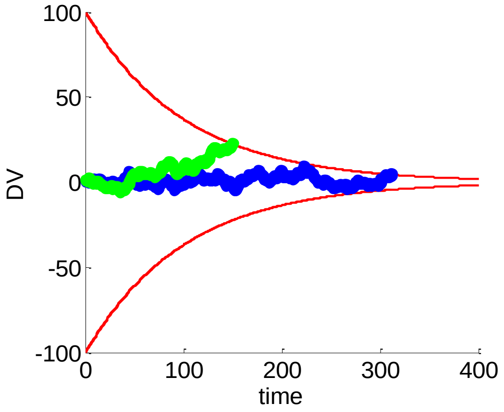

Figure 1: Two exemplar simulations of the DDM with collapsing bounds. The blue and green traces show the decision variable stochastic dynamics (y-axis) as it evolves over time (x-axis). The collapsing bounds are shown in red.

One can see that those trials that effectively take longer to reach the collapsing bound (here: blue trace) correspond to situations in which the DV did (by chance) stay within a limited domain. In contrast, when the DV increases in magnitude (here: green trace), then the bound is hit sooner. This is a direct consequence of the collapsing bounds: the amount of evidence that is required for triggering a choice decreases as elapsed time increases. In other terms, the system triggers the decision despite weak evidence when elapsed time is long enough. As we will be clearer below, this simple phenomenon predicts that CIPC should decrease with RT.

Now Figure 2 below summarizes the results of 200 Monte-Carlo DDM simulations of the same DDM. In these simulations, the drift rate varies randomly at each trial, as well as the initial DV, and we collect the ensuing response times (RT) and final decision variables (DV) when the bound is hit.

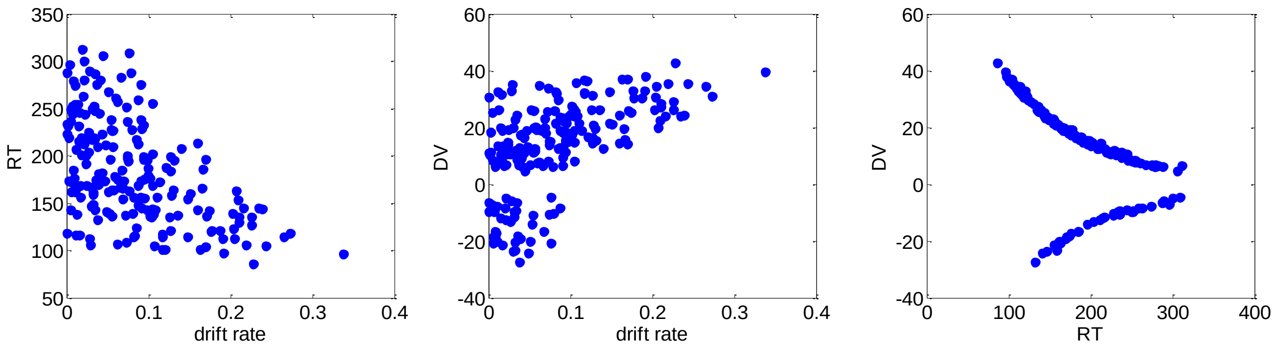

Figure 2: Monte-Carlo simulations of the DDM with collapsing bounds. Left: RT (y-axis) is plotted against drift rate (x-axis). Middle: final DV (y-axis) is plotted against drift rate (x-axis). Right: final DV (y-axis) is plotted against RT (x-axis). Each dot is a simulated trial (here, N=1000). Note that final DV can be negative, which means that the lower bound was hit (despite a positive drift rate).

One can see that, when DR increases, RT decreases (left panel) and the final DV increases (middle panel). Note that -the absolute value of- drift rate is a proxy for choice ease, i.e. inverse choice difficulty. In addition, choice confidence increases with the -the absolute value of- DV (cf. our Results section). This means that under the DDM, when choice difficulty increases, RT increases and choice confidence decreases. In addition, DV decreases when RT increases (right panel), which implies that choice confidence should decrease when RT increases. We note that all these effects have already been demonstrated using behavioral data (De Martino et al., 2012).

Now, under this scenario, the final DV can also be related to CIPC, because it can be seen as the accumulated difference between option values. Here, we measure CIPC in terms of the spreading of

alternative. Recall that the DDM predicts that the choice is determined by which bound (upper or lower) is hit. Therefore, the spreading of alternative (SoA) is given by:

$$SoA = \left(V_{chosen}^{post-choice} - V_{unchosen}^{post-choice}\right) - \left(V_{chosen}^{pre-choice} - V_{unchosen}^{pre-choice}\right)$$
$$= sign\left(DV_{RT}\right) \times \left(DV_{RT} - DV_0\right)$$

where DV_{RT} and DV_0 are the final and initial DV, respectively. The ensuing relationships between CIPC on one hand, and either RT or final DV on the other hand are shown on Figure 3 below:

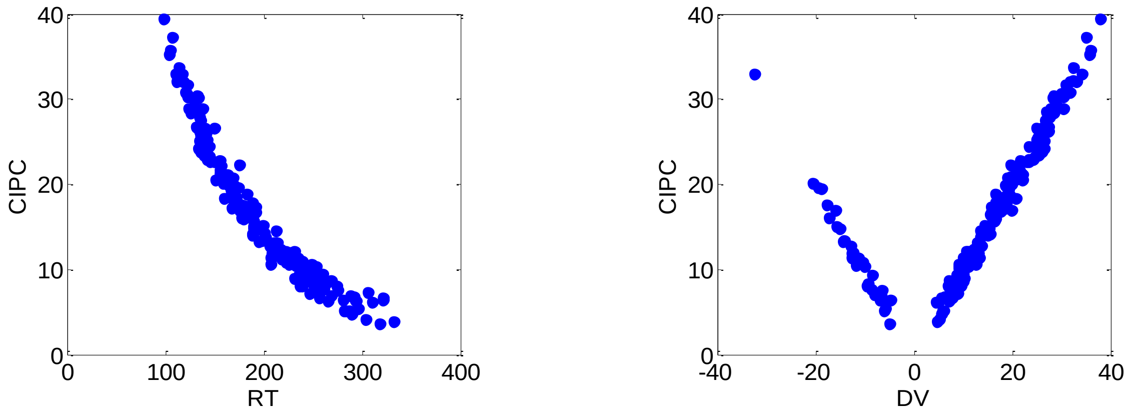

Figure 3: Possible DDM predictions on CIPC. Left: CIPC (y-axis) is plotted against RT (x-axis). Left: CIPC (y-axis) is plotted against final DV (x-axis).

One can see that, under this scenario, CIPC decreases when RT increases, while it increases when choice confidence ($|DV_{RT}|$) increases. Putting this scenario together with our working assumption goes as follows. Increasing choice difficulty does make people deliberate for longer. However, those trials that take the longest are those trials that did not yield much change in the DV (i.e. where the DV stayed within a limited range). In other words, those trials that encounter little or no value spreading take more time because the system has to wait until the bound (i.e. the evidence that is required for triggering a decision) is small enough. In brief, under the DDM with collapsing boundaries, one would expect CIPC to decrease when RT increases.

References

De Martino, B., Fleming, S.M., Garrett, N., and Dolan, R.J. (2012). Confidence in value-based choice. Nat. Neurosci. *16*, 105–110.

Tajima, S., Drugowitsch, J., and Pouget, A. (2016). Optimal policy for value-based decision-making. Nat. Commun. *7*, 12400.
