# Supplementary material for: Choosing what we like vs liking what we choose: How choice-induced preference change might actually be instrumental to decision-making
Source: PLoS One. 2020 May 18;15(5):e0231081. doi: 10.1371/journal.pone.0231081 (PMC7233538; doi:10.1371/journal.pone.0231081)
Supplement: Attachment — Submitted filename: PLOS ONE - response to reviewers.docx [file pone.0231081.s003.docx]

To begin with, we would like to thank the reviewers for their constructive criticism, which, we think, has enabled us to significantly improve our manuscript. We have tried to address each of your comments: you will find below a point by point response (in addition to the changes we made to the previous version of the manuscript, when relevant).

**Response to Reviewer #1:**

*This manuscript describes a study that purports to challenge the mechanistic basis of post-decision cognitive dissonance theory. The tone and language throughout the manuscript needs to be reigned in substantially – this was an incremental behavioral study that shows some evidence that value and choice confidence play a role in influencing changes in valuation quantified after decision-making – it is not a death knell to the theoretical postulates of post-decision cognitive dissonance theory. Some of the hypotheses are framed as novel, but as written, it is not clear that that is the case. Likewise, the discussion frames the report largely in the context of memory research, despite the fact that memory is not measured in the study. These and additional comments are provided below:*

*1. The use of parenthetical statements in the abstract and throughout the introduction is confusing.*

Understood. We have removed some of the parenthetical statements in the abstract and introduction, and restructured others to make them easier to follow.

*2. The writing throughout has a tone that is quite unusual for an academic paper. Just a couple of examples from among: “We take inspiration from..”; “In essence, our experimental design is borrowed from...”*

We have changed these sentences.

*3. In the abstract and MS, the authors need to tone down their claims about whether or not CIPC is adaptive and the implications of CIPC from cognitive and evolutionary perspectives. This extends to claims that CIPC results in irrational biases etc.*

But CIPC *is* worrisome from an evolutionary perspective. For example, in the context of evidence-based decision making, standard cognitive dissonance theory unavoidably predicts the appearance of confirmation and overconfidence biases. This is simply because weak beliefs should be reinforced by subsequent choices, despite the lack of any additional piece of evidence (Allahverdyan and Galstyan, 2014; Navajas et al., 2016). Having re-read the corresponding paragraph in the previous version of our manuscript, we are tempted to think that what eventually made you feel uneasy is that it sounds like the relationship between CIPC and biases is an unorthodox claim, without any authoritative support. We thus have revised the corresponding paragraph in the aim of providing more references to the relevant literature, which we hope will tone down the apparent illegitimacy of this claim (lines 66-79):

This is not to say, however, that the theory of cognitive dissonance reduction has remained unchallenged. The first issue is theoretical in essence. In brief, it is unclear why evolutionary pressure would have favored post-choice cognitive dissonance reduction mechanisms, given that they could eventually induce irrational cognitive biases that have no apparent adaptive fitness (Akerlof and Dickens, 1982; Gilad et al., 1987; Perlovsky, 2013). For example, in the context of evidence-based decision making, standard cognitive dissonance theory predicts the appearance of confirmation and overconfidence biases. This is simply because weak beliefs should be reinforced by subsequent choices, despite the lack of any additional piece of evidence (Allahverdyan and Galstyan, 2014; Navajas et al., 2016). At least in principle, cognitive dissonance reduction may of course have other behavioral consequences that would overcompensate for the adverse selective pressure on cognitive biases. Now if it possessed such adaptive fitness, then it would undoubtedly be expressed in many other animal species. This is, however, an unresolved issue in the existing ethological literature (Egan, Santos & Bloom, 2007; West et al, 2010).

*4. The authors state that they have two hypotheses that are original predictions:*

*“First, the magnitude of CIPC should increase with decision difficulty.” This has been a hypothesis in the context of CIPC for quite sometime. That is why forced choices in the majority of CIPC studies are made between items that are rated as similarly attractive/valuable/desirable etc. At least as written, it is hard to see the distinction between subjective uncertainty and difficulty – they do not seem to be orthogonal factors, but confounded. E.g., This is difficult because I’m not sure which one I’m going to choose.*

We agree that this hypothesis is not new, if one defines decision difficulty in terms of the difference between the items' values. What we wanted to stress in this paragraph, however, was that decision difficulty is actually made of two components: value difference and value uncertainty. More precisely, only our prediction regarding the effect of value uncertainty on CIPC is novel (though it follows the exact same logic as with value difference). This point is tightly related to your next point, which we address in more details below. Now, we acknowledge that our previous wording was ambiguous and we have modified the corresponding paragraph as follows (lines 115-124):

Importantly, our working hypothesis makes two original predictions that deviate from standard post-choice cognitive dissonance reduction theory. Recall that the magnitude of CIPC is known to increase with the absolute difference between pre-choice option values, which is typically taken as a proxy for choice difficulty (Izuma and Murayama, 2013; Sharot et al., 2012). We argue, however, that choice difficulty is better defined in terms of the similarity of value representations. The critical difference is that value representations may be uncertain, i.e. the feeling of liking and/or wanting a given choice option may be imprecise. In other terms, subjective estimates of choice difficulty derive from both value difference and metacognitive judgments about value uncertainty. Our first prediction regards the impact of the latter.

*5. “In particular, we predict that CIPC should increase with subjective uncertainty regarding pre-choice value judgment. This goes against standard post-choice cognitive dissonance reduction theory, because post-choice dissonance should be highest when pre-choice values are similar, with a high judgment certainty.” This second statement doesn’t make sense. CIPC is typically larger when pre-choice values are similar and there is high UNcertainty. Choices are difficult because you are UNcertain which to choose. Prior work also demonstrates effects are greater when you value the items you are choosing about more. It is not clear what is new here.*

Thank you for your comment. We think you are in fact confusing two sorts of uncertainty, namely: uncertainty about valuation and uncertainty about choice. Note that we had used distinct terminology for these distinct concepts throughout our paper: “choice confidence” refers to how confident the decision maker is about the choice itself (ie the comparison of the two values), and “value certainty” refers to how certain the decision maker is about a subjective valuation of an individual item (ie how precise is her feeling of liking and/or wanting the item). Nevertheless, we acknowledge that we should have taken more care in disambiguating these notions. The paragraph that we copied above in response to your previous comment was already modified in the aim of clarifying what we mean with "value uncertainty". Now we agree with you that lower value difference corresponds to lower choice confidence. And we agree that the effect of value difference on CIPC is not new. However, our prediction here refers to value certainty, not value difference (nor choice confidence, which is our second prediction). Our interpretation of standard post-choice cognitive dissonance theory is that a decision maker will feel greater dissonance when he is certain that the individual values are identical:

- Example (high certainty): items A and B are given the same value rating, with a high meta-cognitive certainty judgment; agent chooses A. Because the agent was certain that B was equally good as A, he will experience strong dissonance as he wonders why he chose A over B.
- Example (low certainty): items A and B are given the same value rating, with a low meta-cognitive certainty judgment; agent chooses A. Because the agent really had no idea about the item values, they could a posteriori be quite consistent with her choice and she will thus experience much less dissonance.

We have now modified the corresponding paragraph in our revised manuscript accordingly (see lines 124-134):

In particular, we predict that CIPC should increase with subjective uncertainty regarding pre-choice value judgment. This is because pre-choice value uncertainty raises choice difficulty, which calls for more value reassessment. In contrast, standard post-choice cognitive dissonance reduction theory predicts that post-choice dissonance should be highest when pre-choice values were *a priori* certainly equal, i.e. for choices with minimal value distance and maximal value certainty. Second, we predict that CIPC should positively correlate with choice confidence, controlling for the impact of decision difficulty. This is because, under our hypothesis, CIPC indirectly signals a successful improvement of choice confidence, due to reassessed values spreading apart. In contrast, standard dissonance reduction theory would posit that choices made with low confidence should trigger the strong aversive dissonance feelings that eventually lead to CIPC.

*6. Is there a significant difference in the age across the two groups? If so, all analyses must control for age.*

There is, indeed, a statistically significant difference in the age across the groups. The difference is caused by the presence of several more older adults in the control group than in the main group, which had an age distribution typical for this type of study. We thus invited 12 additional participants to perform the experiment, in order to match the ages of the RCR and RRC groups. We have re-performed the same analyses, and updated the report of our summary statistics in the revised version of the manuscript. In brief, the resulting group comparisons remain qualitatively unchanged.

*7. There are infinity symbols in many of the confidence intervals.*

Some of our statistical tests were one-sided rather than two-sided, because we had *a priori* theoretical beliefs about the direction of the relationship that we expected (the end of confidence intervals of one-sided statistical tests are always unbounded).

*8. “Recall that, under our hypothesis, effort allocation during the decision process is expected to refine internal value representations up until a target level of confidence is met and the decider commits to a choice.” Given that effort allocation is a construct of interest, response time should be reported and analyzed? Even this cannot determine that internal value representations are refined up until a target level of confidence is met. The authors need to reframe this construct in the context of what they are objectively measuring.*

You are right: we should not mention effort at this point, given that we do not have any clear empirical measure of it. We have thus simplified this sentence as follows (lines 226-228):

Recall that, under our hypothesis, the deliberation that takes place during the decision process is expected to cause a refinement of internal value representations until a target level of choice confidence is met and the decider commits to a choice.

Note that we have proofread our Introduction and Results sections to remove any mention to effort. We now only mention effort in the Discussion section, when comparing our results to the literature showing that activity in the anterior cingulate cortex (ACC) during the choice process predicts the magnitude of CIPC (lines 353-359):

Note that current neurocomputational theories of ACC suggest that it is involved in controlling how much mental effort should be allocated to a given task, based upon the derivation of the so-called "expected value of control" (Musslick et al., 2015; Shenhav et al., 2013). This is highly compatible with our results, under the assumption that pre-choice value reassessment is a controlled and effortful process that trades mental effort against choice confidence. We will pursue this computational scenario in subsequent publications.

*9. The discussion of memory steps beyond the data collected, yet seems to be the primary focus of the discussion. E.g., “Our results apparently contradict the recent finding that CIPC only occurs for choices where the agent is later able to recall which option was chosen and which was rejected (Salti et al, 2014).” The authors do not test memory, thus their results cannot contradict these findings. The majority of this entire paragraph seems inconsistent with the data that were actually collected.*

Although we do not study the relationship between memory and CIPC, we still think this line of research is quite relevant for discussing our results. This is because the dependence of CIPC on choice memory has been taken as indirect evidence that CIPC occurs post-choice (which we challenge here). This is why we feel that we cannot simply ignore this result, but rather need to provide alternative interpretations that would make it consistent with our pre-choice interpretation of CIPC. We have tried to modify the corresponding paragraph to make the connection between this work and ours more clear (lines 316-324):

Our results apparently contradict the recent finding that CIPC only occurs for choices where the agent is later able to recall which option was chosen and which was rejected (Salti et al, 2014). This is because remembering choices has no causal role under our pre-choice value refinement hypothesis. This contrasts with standard cognitive dissonance reduction theory, where post-choice option re-evaluation requires the memory trace of relevant choices. This interpretation is compatible with the observation that, when re-evaluating items after the choice, activity in the hippocampus discriminates between remembered and non-remembered choices (Chammat et al, 2017). These two studies thus provide apparent evidence that CIPC occurs after the choice has been made. However, (...)

In fact, we also took this revision as an opportunity to discuss our results in the light of another line of research from Tali Sharot's group, which also provides evidence for post-choice CIPC. We thus added the following paragraph to the Discussion section (lines 360-377):

Nevertheless, we consent that CIPC may be driven by both pre-choice value reassessment and post-choice cognitive dissonance reduction mechanisms. The quantitative contribution of the latter effect, however, may have been strongly overestimated. In our view, this is best demonstrated, though perhaps unintentionally, in the results of the “blind choice” study from Sharot and colleagues (Sharot et al., 2010). Here, participants rated items both before and after making a blind choice that could not be guided by pre-existing preferences (because the items were masked). Critically, although blind choice precludes any instrumental value refinement process, preferences were altered after the choice. Interestingly, the effect size is rather small, i.e. the ensuing CIPC magnitude was estimated to be around 0.07 ± 0.03. This is to be compared with the CIPC magnitude of the RCR and RRC conditions in two other studies by the same authors (Sharot et al., 2009, 2012), namely: 0.38 ± 0.08 (RCR condition, with non-blind choices) and 0.11 ± 0.06 (RRC condition, which was not included in the “blind choice” study). In other terms, CIPC under blind choice is smaller than the apparent CIPC that unfolds from the known statistical confounds of the free choice paradigm. Note that if this had not have been the case, then post-choice CIPC cognitive reduction effects would dominate and we would not have confirmed our predictions. Taken together, this series of studies clearly supports the notion that option re-evaluation occurs, at least partly, at the time of the decision.

**Reponse to Reviewer #2:**

*Choice induced preference change (CIPC) is a fascinating subject as a behavioral manifestation of the fundamental process of decision making. The problem has been around for almost 60 years. Yet how this phenomenon occur remains a mystery. One of the important questions about CIPC is when the processes causing preference change occur. The authors tried to address this question with a new behavioral paradigm, which included confidence reports at preference rating and choice between rated objects. The authors’ hypothesis is that the metacognitive processes occurring during a decision making induce the preference change. I have a sympathy to this claim itself. But the evidence presented in this study cannot prove this hypothesis.*

*Major Point*

*The processes related to the confidence report can occur after the decision making. Thus, whatever the relationship between the confidence reports and CIPC cannot prove the author’s claim.*

We thank you for providing us with an opportunity for disambiguating a critical aspect of our work.

If we understand correctly, you are considering the possibility that CIPC may have occurred after people commit to a choice, but before they get a feel for how confident they are about their choice. This sounds paradoxical, in the sense that experiencing cognitive dissonance in this context simply means feeling uneasy about one's choice, i.e. lacking confidence about it. We acknowledge that this is, at least in principle, possible. However, your reasoning cannot apply to our interpretation of the impact of value certainty. Recall that we probe metacognitive judgments about value certainty before the choice, using rating scales at the time when each item is presented (right after first-order value judgments). Therefore, the relationship between CIPC and value certainty that we disclose empirically cannot derive from metacognitive processes that occur after the choice has been made. We have included a novel paragraph in the Discussion section of this manuscript to summarize these points (lines 305-315):

Note that one may challenge our interpretation of the observed relationship between confidence and CIPC, in terms of evidence against post-choice CIPC. For example, one may argue that CIPC might occur after people commit to a choice, but before they get a feel for how confident they are about that choice. This sounds paradoxical however, in the sense that experiencing cognitive dissonance in this context simply means feeling uneasy about one's choice, i.e. lacking confidence about it. In any case, this line of reasoning cannot apply to the observed impact of value certainty on CIPC. Recall that we probe metacognitive judgments about value certainty before the choice, using rating scales at the time when each item is presented (immediately after first-order value judgments). Therefore, the relationship between CIPC and value certainty that we disclose empirically cannot derive from metacognitive processes that occur after the choice has been made.

Note that, in the aim of disambiguating the nature of our working hypothesis, we also have partly rewritten the paragraph in the Introduction section where we refer to the literature that hints at the fact that CIPC may occur during the decision process (lines 95-107):

Interestingly, recent neuroimaging evidence suggests that, in the context of typical free-choice paradigms, preference changes occur *during* the decision, not *after* it (Jarcho, Berkman, & Lieberman, 2010; Colosio et al, 2017). Recall that people are reluctant to make a choice that they are not confident about (De Martino et al, 2013). But envisioning competing possibilities during a choice provides a new context that highlights the unique aspects of the alternative options (Tversky & Thaler, 1990; Lichtenstein & Slovic, 2006; Warren, McGraw, & Van Boven, 2011). In turn, the act of choosing may change preferences by reappraising aspects of choice options that may not have been considered thoroughly before (Sharot et al., 2009). In other words, preferences may change during deliberation in order to facilitate the choice process (Simon, Stenstrom & Read, 2015; Simon, Krawczyk & Holyoak, 2004; Simon & Holyoak, 2002). This is important, because it allows for the possibility that preference changes may be instrumental for the process of decision making, which would resolve most theoretical concerns.

We hope that you will find our response to your comment satisfactory.
