# Supplementary material for: Choosing what we like vs liking what we choose: How choice-induced preference change might actually be instrumental to decision-making
Source: PLoS One. 2020 May 18;15(5):e0231081. doi: 10.1371/journal.pone.0231081 (PMC7233538; doi:10.1371/journal.pone.0231081)
Supplement: Attachment — Submitted filename: Response to Reviewers.docx [file pone.0231081.s004.docx]

**Response to Reviewer #1:**

*The authors failed to address key issues raised in the prior review.*

*- From the most basic perspective, the methods utilized in this paper cannot discriminate mechanisms of pre-choice value refinements from post-choice cognitive dissonance reduction. Certainty about choice measures are obtained after the choice is made. There is no way to disentangle what happens during the decision to what happens after, as data about certainty were only collected after the decision.* *Moreover, the authors state: “Therefore, the relationship between CIPC and value certainty that we disclose empirically cannot derive from metacognitive processes that occur after the choice has been made.” As noted in the prior review, the authors have no data to support this and other assertions about the cognitive processes engaged during and after decision-making. Response times could perhaps provide some empirical support for their assertions, however, they failed to include these data in their revision.*

We respectfully disagree with you. We think that your comment may be reflecting a misunderstanding, which we were unable to prevent during the previous review round. In brief, you are confusing choice confidence (which was collected after the decision) with reports of value certainty (which were collected before the decision). This distinction is important, and relates to your next point (which we think is also driven by the same confusion). For the sake of clarification, let us recall our experimental paradigm. First, participants were asked to rate each item's value and report their level of uncertainty regarding this value rating. We refer to these as "pre-choice value" and "pre-choice value certainty", respectively. Second, participants are asked to chose between pair of items and report their confidence about this decision. We refer to the latter as "choice confidence". The third session is identical to the first one, and we refer to the corresponding measures as "post-choice value" and "post-choice value certainty". We have modified the manuscript in the aim of making the distinction between choice confidence and pre-choice value certainty as clear as possible:

- Introduction (l.131-140):

We note that pre-choice value uncertainty is collected for each item, and is different from choice confidence (which we collect for each choice). In particular, we predict that CIPC should *increase* with pre-choice value uncertainty. This is because pre-choice value uncertainty raises choice difficulty, which calls for more value reassessment. The prediction of standard post-choice CDRT regarding the impact of pre-choice value uncertainty are less clear. But if anything, we would argue that post-choice dissonance should be highest when pre-choice values were certainly equal, i.e. for choices with minimal value distance and maximal value certainty. Thus, under post-choice CDRT, CIPC should rather *decrease* with pre-choice value uncertainty.

- Introduction (l.143-146):

In contrast, post-choice CDRT would posit that choices made with low confidence should trigger the strong aversive dissonance feelings that eventually lead to CIPC. Thus, under post-choice CDRT, CIPC should *decrease* with choice confidence. These predictions are summarized in Figure 1 below.

[See the copy of Figure 1 below]

- Methods (l.203-209):

Participants had to respond to the question, “Does this please you?” using a horizontal Likert scale (from “not at all” to “immensely”) to report their subjective valuation of the item. Participants then had to respond to the question, “Are you sure?” using a vertical Likert scale (from “not at all” to “immensely”) to indicate their level of subjective uncertainty regarding the preceding value judgment. We refer to this as a *value uncertainty rating* (which is not to be confounded with *choice confidence ratings*, see below).

- Methods (l.220-224):

Participants had to respond to the question, “What do you prefer?” using the left or right arrow key. Participants then had to respond to the question, “Are you sure about your choice?” using a vertical Likert scale to report their level of confidence in the preceding choice. We refer to the latter as a *choice confidence rating* (not to be confounded with *value uncertainty ratings*, see above).

Having said this, we acknowledge that we may have worsen the consequence of this confusion, by not highlighting clearly the goal of our experiment. The classical explanation for CIPC is cognitive dissonance reduction theory (hereafter: CDRT). Tantamount to this theory is the notion that CIPC occurs after the choice has been made, as in “I chose (rejected) this item, so I must have liked it better (worse) than the other one”. However, we are NOT trying to provide evidence for the fact that CIPC occurs during (as opposed to after) the decision. Rather, we are trying to provide evidence in favor of another interpretation of CIPC, namely: that people reassess option values until they reach a satisfactory level of confidence (at which point they commit to a choice). Or more precisely, we are trying to show that CIPC is in fact expected, without referring to any post-choice cognitive dissonance reduction mechanism. It turns out that our working hypothesis makes two quantitative predictions that deviate from post-choice CDRT, which we validate empirically. We quickly discuss these predictions below. The critical point is that these predictions are not about the timing of CIPC: they relate to the basic cognitive mechanisms that cause CIPC under the two theories. We tend to think that the criticisms related to CIPC timing may eventually have been triggered by our clumsy comparison of these two theories. In particular, in the Discussion section, we say that our working assumption could be viewed as some sort of intra-choice cognitive dissonance reduction mechanism. We have now removed this -and similar- unfortunate mentions of temporal aspects of CIPC (during versus after choice) from the manuscript. And we have made additional modification with the aim of clarifying as much as possible the goal of our study (in particular in the Abstract and Introduction sections):

- Abstract (l.30-37):

The results confirm our predictions and provide evidence that many features of CIPC may be explained without invoking post-choice cognitive dissonance reduction explanation.

- Introduction (l. 120-122):

In brief, post-choice cognitive dissonance reduction theory (hereafter: post-choice CDRT) states that people come to liking what they have chosen. We rather suggest, somewhat trivially, that they may simply be choosing what they have come to liking.

- Introduction (l.151-156):

That CDRT predicts the above three-way relationship between CIPC, choice confidence and value uncertainty, was, to the best of our knowledge, never stated until now. It is thus legitimate to criticize this prediction. We will briefly comment on this in the Discussion section. At this point, suffice it to say the primary goal of this study is to ask whether CIPC can be explained without invoking CDRT. We reason that it would be the case, should CIPC varies according to the prediction made under our working (alternative) hypothesis.

- Discussion (l.313-319)

According to standard post-choice CDRT, choices made with low confidence trigger strong aversive dissonance feelings that are resolved by retrospectively matching internal value representations to the choice. We would rather say that no choice commitment is made until internal value representation refinements allow choice confidence to reach a satisfying (non-aversive) level. Our results show that CIPC varies as predicted by the latter scenario. This is important, because this implies that CIPC may be explained without referring to CDRT.

- Discussion (l.330-336):

In any case, we are not trying to provide evidence for the fact that CIPC occurs during (as opposed to after) the decision. Rather, we are trying to provide evidence in favor of another interpretation of CIPC, namely: that people reassess option values until they reach a satisfactory level of confidence (at which point they commit to a choice). Or more precisely, we are trying to show that CIPC is in fact expected, without referring to any post-choice cognitive dissonance reduction mechanism.

We note that we don't see how reaction time data could, at this stage, help us discriminate between these two scenarios, given that, to the best of our knowledge, none of them makes any clear related prediction.

*- The authors contradict themselves when positing what standard theories predict. Here is one example: “In contrast, standard post-choice cognitive dissonance reduction theory predicts that post-choice dissonance should be highest when pre-choice values were a priori certainly equal, i.e. for choices with minimal value distance and maximal value certainty.” ... “In contrast, standard dissonance reduction theory would posit that choices made with low confidence should trigger the strong aversive dissonance feelings that eventually lead to CIPC.” First they state that CIPC would be highest when pre-choice are “certainly equal” – which I take to mean there is high confidence in their value. Second they say that CIPC would be highest when pre-choices are “made with low confidence” – which I take to mean there is low confidence in their value. These assertions continue to be problematic.*

This comment is, in our opinion, entirely due to the confusion we mentioned before. We, in fact, do not make any contradicting statements: they are simply about either "pre-choice value certainty" (first statement) or about “choice confidence” (second statement). But here again, we acknowledge that we did not provide a way to picture our reasoning in a clear and concise manner. We thus have modified our manuscript to clarify the derivation of these predictions as much as possible (l.133-146):

In particular, we predict that CIPC should *decrease* with pre-choice value certainty. This is because pre-choice value certainty lowers choice difficulty, which de-motivates value reassessment. The prediction of standard post-choice CDRT regarding the impact of pre-choice value certainty are less clear. But if anything, we would argue that post-choice dissonance should be highest when pre-choice values were certainly equal, i.e. for choices with minimal value distance and maximal value certainty. Thus, under post-choice CDRT, CIPC should rather *increase* with pre-choice value certainty. Second, we predict that CIPC should *positively* correlate with choice confidence, controlling for the impact of decision difficulty. This is because, under our hypothesis, CIPC indirectly signals a successful improvement of choice confidence, due to reassessed values spreading apart. In contrast, post-choice CDRT would posit that choices made with low confidence should trigger the strong aversive dissonance feelings that eventually lead to CIPC. Thus, under post-choice CDRT, CIPC should *decrease* with choice confidence. These predictions are summarized in Figure 1 below.

Under the advice of the Editor, we also have now included a figure that summarizes the predicted relationship between CIPC and meta-cognitive judgments, under either post-choice CDRT, or under our assumption. It is reproduced below:

Figure 1: Comparison of predicted relationships between meta-cognitive judgments and CIPC. Here, we summarize the main predictions of how CIPC relate to meta-cognitive judgments under post-choice CDRT (left panel), and under our hypothesis (right panel). Blue bars depict putative partial correlations between CIPC on the one hand, and absolute value difference, pre-choice value certainty and choice confidence, on the other hand. One can see that, except for the effect of absolute value difference, our hypothesis make qualitatively distinct predictions from post-choice CDRT.

We hope that this Figure (and the corresponding modifications of the main text) will clarify our reasoning and prevent this kind of confusion.

*- The authors do not measure memory. As noted in the prior review, the paragraph claiming the results contradict recent memory-based findings steps well beyond the data. It should be removed.*

Recall that we do not try to provide evidence for the timing of CIPC (during or after choice). Rather, we aim at showing that CIPC is in fact expected without any cognitive dissonance reduction. Now it turns out that memory-based findings are important, because they provide indirect evidence for CDRT. In other terms, if memory of the choice is really necessary for CIPC to occur, then our alternative interpretation is simply wrong. This is why we think discussing memory-based findings is relevant for our work. Here again, we have modified this paragraph of the Discussion section to make this clearer (l.342-351):

Let us now discuss our results in the context of the existing literature affording evidence in favor of post-choice CDRT. In particular, CIPC was recently shown to only occur for choices where the agent is later able to recall which option was chosen and which was rejected (Salti et al, 2014). This is somewhat problematic because remembering choices has no causal role under our pre-choice value refinement hypothesis. This contrasts with standard CDRT, where post-choice option re-evaluation requires the memory trace of relevant choices. The latter interpretation is compatible with the observation that, when re-evaluating items after the choice, activity in the hippocampus discriminates between remembered and non-remembered choices (Chammat et al, 2017). These two studies thus provide apparent evidence for post-choice CDRT. However,...

*- No statistics are provided regarding age and sex distributions across conditions.*

We think that this is a slightly unfair comment, given that we included 13 more participants in the second review round to address your previous comment regarding potential age/gender group differences (we did provide age and gender summary statistics for the two groups). We confirm that there is no significant difference in either age or gender between the two groups. For the sake of completeness, we now include p-values for the statistical tests in the modified manuscript (l.178-179):

These groups did not show any significant difference in terms of age (p=0.06) or gender (p=.27).

*- The authors fail to state that one-sided statistical tests were used in some analyses.*

We used one-sided statistical tests whenever our hypotheses were one-sided (cf., e.g., Figure 1 above), and the report of one-sided confidence intervals make this very concrete. Nevertheless, we now have modified our Results section, explicitly stating that we use one-sided test each time we use them.

**Response to Reviewer #2:**

*The authors addressed my concern about the confidence reports after the decision by stating that the other metacognitive measure, value certainty. Value certainty is recorded before the choice. But does this serve as metacognitive measure of intra-choice process? I commented on this measure (reports of choice confidence) because this is supposed to reflect the intra-choice process. The pre-choice measures of certainty about value judgments have limitations in estimating the intra-choice processes because the choice process has not happened yet. I recommend that the authors should be careful about the distinctions of the two metacognitive measures and clearly state the limitations.*

We are relieved to see that you noticed the distinction between choice confidence and pre-choice value certainty reports. We agree that it may indeed induce unfortunate confusion in the mind of our readers. We have made further modifications to the manuscript to make this distinction as clear as possible. We of course acknowledge the limitations of these measures in our modified manuscript. But let us reiterate here our response to a related point by Reviewer #1. We are not trying to provide evidence for the fact that CIPC occurs during (as opposed to after) the decision. Rather, we are trying to provide evidence in favor of an interpretation of CIPC which is distinct from CDRT, namely: that people reassess option values until they reach a satisfactory level of confidence (at which point they commit to a choice). Or more precisely, we are trying to show that CIPC is in fact expected, without referring to any post-choice cognitive dissonance reduction mechanism. It turns out that our working hypothesis makes two quantitative predictions that deviate from standard post-choice CDRT. These predictions regard the relationship between CIPC and meta-cognitive judgments. For the purpose of validating these predictions, and therefore showing that CIPC behaves as expected, these meta-cognitive measures are, in our opinion, reliable enough. In addition to the above changes, we have modified the corresponding paragraph of the Discussion (l.330-340):

In any case, we are not trying to provide evidence for the fact that CIPC occurs during -as opposed to after- the decision (we discuss the related literature below). Rather, we are trying to provide evidence in favor of another interpretation of CIPC, namely: that people reassess option values until they reach a satisfactory level of confidence (at which point they commit to a choice). Or more precisely, we are trying to show that CIPC is in fact expected, without referring to any post-choice cognitive dissonance reduction mechanism. It turns out that our working hypothesis makes two quantitative predictions that deviate from standard post-choice CDRT. These predictions regard the relationship between CIPC and meta-cognitive judgments. For the purpose of validating these predictions, and therefore showing that CIPC behaves as expected, these meta-cognitive measures are, in our opinion, reliable enough.

We hope that this (and the other changes we have made to our manuscript) will clarify our objective and methods.
