# Supplementary material for: Choosing what we like vs liking what we choose: How choice-induced preference change might actually be instrumental to decision-making
Source: PLoS One. 2020 May 18;15(5):e0231081. doi: 10.1371/journal.pone.0231081 (PMC7233538; doi:10.1371/journal.pone.0231081)
Supplement: Attachment — Submitted filename: Response to Reviewers.docx [file pone.0231081.s005.docx]

**Editor's comment**

*After careful consideration and consultation between editorial members, we feel that your manuscript has merit but does not fully meet PLOS ONE’s publication criteria as it currently stands. More specifically, as pointed out by Reviewer #1, RT data could provide essential information about proving or disproving alternative hypotheses/models and therefore, analyses of RT data should be provided and discussed. In case RT data were not collected, this should be mentioned as a limitation of the study. We also feel that some of the statements about debunking cognitive dissonance theory based on the results of one experiment should be toned down.*

We thank you for giving us another opportunity to improve our manuscript. As you will see below, we have responded to all remaining comments. In particular, we now report and discuss a statistical analysis of the relationship between CIPC and RT data. We have also toned down most of our claims about debunking cognitive dissonance theory. The associated modifications are highlighted using Word's Track Changes in the modified manuscript.

**Response to Reviewer #1:**

*Reviewer #1: The authors were able to address some, but not all, issues raised in prior reviews. Further clarification shed light on other issues that need to be addressed.*

*1. The authors suggest that change in value occurs during decision-making. They state in their abstract “…that CIPC is at least partially- due to the refinement of option value representations that occurs while people are pondering about choice options. For example, contemplating competing possibilities during a choice may highlight aspects of the alternative options that were not considered before. In the context of difficult decisions, this would enable people to reassess option values until they reach a satisfactory level of confidence.” And then later: “Critically for our theory, the more difficult the decision, the more deliberation and potential reassessment of value representations, the more likely a change of mind and the related CIPC.” And then after that: “Recall that, under our hypothesis, the deliberation that takes place during the decision process is expected to cause a refinement of internal value representations until a target level of choice confidence is met and the decider commits to a choice.” Similar statements are made at various other points in the manuscript.*

*As noted in both prior reviews – if reassessing values during decision-making is a critical feature driving CIPC, then response time is an important data point that could corroborate the authors’ assumptions. Namely – the more people are “contemplating competing possibility”, the longer the decision should take to make (i.e., longer the RT). This should, in turn relate to change in values. Failure to report RT because it was not collected is at minimum a critical limitation of the study. If RT was collected and does not relate to change in value, this is an important point that must be discussed as it is inconsistent with the explanation the authors describe throughout the manuscript. I disagree with the authors’ conclusion that RT is irrelevant to supporting the contentions they explicitly note above and throughout the manuscript. If the authors have misspoken throughout the manuscript about the cognitive mechanisms they believe are implicated in CIPC, then those inaccuracies need to be corrected.*

We understand your concern and share it to some extent. We agree that, to the extent that it can be thought of as a proxy for how much people ponder their decision, RT data may provide some additional piece of evidence for our pre-choice interpretation of CIPC. The reason why we are not entirely enthusiastic about including analyses of RT data is that the actual prediction one would draw regarding its relationship to CIPC actually depends upon the covert cognitive/computational mechanism that eventually trigger the decision (and therefore determine RT). One possibility is the one you highlight, which essentially states that CIPC increases with RT, because more deliberation should lead to more value changes. But this prediction actually relies upon an implicit assumption regarding the covert mechanism that triggers decisions. In brief, this prediction holds under the assumption that decision deliberation stops when some decision variable (e.g. value spread) eventually reaches some sort of preset threshold. But other alternative decision mechanisms may be possible. For example, deliberation may also stop when enough effort has been invested. In this case, difficult decisions would yield long deliberations, but may encounter little or no value spreading. In this case, choices with long RT are those made with low confidence and small CIPC. Both scenarios predict that CIPC should correlate positively with choice confidence (though for slightly different reasons). But they differ with respect to the sign of the predicted relationship between CIPC and RT. In our view, this makes analyses of RT data even more difficult to grasp than the results we already report in our manuscript. Nevertheless, we have tested whether any such relationship holds in our data. We eventually found a significant negative correlation between RT data and CIPC, as predicted by the second scenario above. For the sake of completeness, we have decided to report this results in the following additional paragraph of the modified Discussion section (l. 327-351):

Given the critical impact of decision deliberation on CIPC (under our hypothesis), can we make predictions regarding the relationship between response time (RT) and CIPC? Recall that we hypothesize that value reassessment during decision-making is a critical determinant of CIPC. This might tempt one to predict that longer RT should lead to greater CIPC. The logic of this idea would be that difficult decisions cause people to deliberate longer, which in turn allows for larger changes in value estimates. But in fact, this reasoning actually relies upon an implicit assumption regarding the covert mechanism that triggers decisions. In brief, this prediction holds under the assumption that decision deliberation stops when some decision variable (e.g. value difference) eventually reaches some preset threshold. This is, in fact, what most accumulation-to-bound models (e.g., drift-diffusion decision models) typically assume (Ratcliff et al., 2016). But other alternative mechanisms may be possible. For example, deliberation may also stop when enough effort has been invested. In this case, difficult decisions would yield long deliberations, but may encounter little or no value spreading. In this case, choices with long RT are those made with low confidence and small CIPC. Both mechanisms predict that CIPC should correlate positively with choice confidence (though for slightly different reasons). But they differ with respect to the sign of the predicted relationship between CIPC and RT. Importantly, standard post-choice CDRT is, to our knowledge, agnostic about any such relationship. In our data, the correlation between CIPC and RT turns out to be significant and negative, even when controlling for pre-choice decision variables (mean correlation = -0.1236, p<0.001). Thus, this may be seen as an additional piece of evidence in favor of the idea that CIPC is, at least partially, driven by value reassessment that happens during the decision. We note that other covert decision control mechanisms may in fact be considered. A proper comparison of such processes would require an exhaustive evaluation of their computational properties (Lee and Daunizeau, 2019). We intend to pursue this in subsequent publications.

*2. Grammar problems in this sentence: “In brief, post-choice cognitive dissonance reduction theory (hereafter: post-choice CDRT) states that people come to liking what they have chosen. We rather suggest, somewhat trivially, that they may simply be choosing what they have come to liking.”*

Thank you for spotting this. We have now corrected it as follows (l. 120-122):

In brief, post-choice cognitive dissonance reduction theory (hereafter: post-choice CDRT) states that people come to like what they have chosen. We rather suggest, somewhat trivially, that they may simply be choosing what they have come to like.

*3. I appreciate the inclusion of the new figure 1. Unfortunately, it was not as helpful as it could have been. This may be due to insufficient description/labeling of the Y axis (is it appropriate to conceptualize this more simply as partial correlation with SoA?) and lack of clarity about what value certainty and choice confidence represent (selected – unselected items; unselected – selected; something else?).*

We appreciate your comment. We have now modified the figure caption as follows (l. 590-597):

**Figure 1: Comparison of predicted relationships between meta-cognitive judgments and CIPC.** Here, we summarize the main predictions of how CIPC relate to meta-cognitive judgments under post-choice CDRT (left panel), and under our hypothesis (right panel). Blue bars depict putative partial correlations between CIPC on the one hand, and absolute pre-choice value difference (between options), pre-choice value certainty (averaged across options), and choice confidence, on the other hand. One can see that, except for the effect of absolute value difference, our hypothesis make qualitatively distinct predictions from post-choice CDRT.

*4. It’s great that the authors collected more data to deal with the apparent age difference across groups. I also appreciate that the authors provided statistics about sex and age differences across conditions – I am not entirely clear on why they deemed such a request as unfair as it is standard practice in most journals to report statistics on demographic features of samples. Because p = .06 is still a marginal difference, this issue should be noted as a limitation in the discussion section. Additionally, please demonstrate that results are significant after controlling for age.*

Unfortunately, we can't control for age because we didn't collect demographic data during the first part of the experiment. More precisely, information about participant ages was stored by the tech staff without any connection to the collected behavioral data. This means that we only have anonymous statistics about the two groups (except for the newly acquired subjects, but they all belong to the RRC control group). Nevertheless, we have added the following paragraph in the modified Discussion section (l. 350-366):

As a side note, we acknowledge that our group comparison (cf. RCR group versus RRC control group) suffers from the typical limitations of between-subject designs. In particular, and although we did not find any significant age difference between the two groups, trivial age differences may still in principle confound our comparison. If this was the case, then one may challenge the validity of our control RRC group. Recall that this control is important for demonstrating that CIPC exists above and beyond the expected statistical bias reported in Chen and Risen (2010). Here, we used the RRC control group to show that the magnitude of the statistical relationship between CIPC and metacognitve judgments (in the RCR group) exceeds what can be expected from a situation where decision processes cannot interfere (cf. RRC group). Given that virtually nothing is known about the effect of age on CIPC (Cooper and Feldman, 2019), it is thus theoretically possible that our observed group differences may in fact be driven by some variant of Chen and Risen's statistical artifact. However, Chen and Risen's reasoning does not apply straightforwardly to predictions regarding statistical relationships between CIPC and metacognitive judgments. In addition, such age confound is a priori unlikely, given that many studies previously already reported a significant difference between RRC and RCR groups or conditions (Salti et al, 2014; Coppin et al, 2013; Coppin et al, 2012; Sharot et al, 2012).

*5. Given that the authors are testing competing models that have different predicted directionality (positive vs. negative correlations, if Figure 1 is in fact depicting partial correlations with SoA), the use of one-sided tests in some of their most critical analyses are not appropriate. For example, results from a one-sided analysis are purported to demonstrate: “The latter findings support our hypothesis, and are incompatible with classical post-choice CDRT.”*

Well, we respectfully disagree with you here. It is precisely because our hypotheses are directional that we use one-sided tests. Recall that if p is the p-value for a given one-sided test, then 1-p is the p-value for the opposite one-sided test. Therefore, if we test our one-sided assumption (H1: effect>0 and therefore H0:effect<0), and show that, e.g. p=0.01 (which validates the assumption H1), then we know that the opposite assumption (H1: effect<0 and H0:effect>0) would have got a p=0.99 p-value (i.e. in that case one could not reject the null). Alternatively, if we had performed a two-sided hypothesis test (H0: effect=0), we would have got a p=0.02 p-value and this could either be due to a positive or a negative effect (ie, this could not be used as evidence for or against any of the directional assumptions).

*6. “We then asked whether post-choice ratings better predict choice (and choice confidence) than pre-choice ratings. First, we performed another logistic regression of paired choices, this time against the difference in post-choice value ratings (ratings #2). The ensuing choice prediction accuracy is higher than with pre-choice value ratings (accuracy = 0.787, 95% CI [0.770, 0.804], accuracy gain = 0.103, 95% CI [0.082, 0.124], p<0.001) (Figure 3).” The second sentence does not mention anything about pre-choice ratings. Clarify. Same comment on the next analysis described in the “Second, we regressed choice confidence…” part.*

We acknowledge that the way we reported our statistical results may have been ambiguous. We have modified the corresponding paragraph as follows (l. 271-278):

The ensuing balanced prediction accuracy is significantly higher than with pre-choice value ratings: prediction accuracy given post-choice ratings = 0.787, 95% CI [0.770, 0.804], prediction accuracy gain (post-pre) = 0.103, 95% CI [0.082, 0.124], p<0.001 (Figure 3). Second, we regressed choice confidence, this time against *post*-choice absolute value difference and mean value certainty. The ensuing amount of explained variance is higher than with pre-choice ratings: R^2^ given post-choice ratings = 0.245, 95% CI [0.209, 0.281], R^2^ gain (post-pre) = 0.022, 95% CI [0.001, 0.042], p=0.02 (Figure 3).

*7. The traditional result is that the difference in value for two items in a pair increases when the pair are similar in pre-choice value. As described in the results section: “ "spreading of alternatives" (SoA), calculated as the mean difference in value rating gains between chosen and unchose items (SoA = [rating#2-rating#1]chosen - [rating#2-rating#1]unchosen).” It is unclear if this is the effect described here: “As expected, a random effect analysis on the ensuing parameter estimates shows that SoA significantly decreases with the absolute difference in pre-choice value ratings.” Is it that SoA decreases as the absolute difference in pre-choice value ratings increase? Likewise, description of directionality is needed for results that describe SoA in relation to pre-choice value certainty ratings (i.e., SoA decreases when pre-choice value certainty ratings are higher/lower) and choice confidence (SoA increases with higher/lower levels of choice confidence).*

Yes, this analysis simply reproduces this very well established result. We have corrected our wording to make this entirely clear (l. 293-299):

As expected, a random effect analysis on the ensuing parameter estimates shows that SoA significantly decreases when the absolute difference in pre-choice value ratings increases (GLM beta = -0.295, 95% one-sided CI [-∞, -0.265], p<0.001). More importantly, we found that SoA significantly decreases when pre-choice value certainty ratings increases (GLM beta = -0.065, 95% one-sided CI [-∞, -0.036], p<0.001) and significantly increases when choice confidence increases (GLM beta = 0.200, 95% one-sided CI [0.170, ∞], p<0.001).
