# Supplementary material for: Choosing what we like vs liking what we choose: How choice-induced preference change might actually be instrumental to decision-making
Source: PLoS One. 2020 May 18;15(5):e0231081. doi: 10.1371/journal.pone.0231081 (PMC7233538; doi:10.1371/journal.pone.0231081)
Supplement: Attachment — Submitted filename: Response to Reviewers.docx [file pone.0231081.s006.docx]

**Editor's comment**

*Another Associate Editor and I have now read your response to the final reviewer's criticisms/comments. Overall, we think that you have addressed all issues except one (about the relationship between RT and CIPC). As a result, we feel that your manuscript has merit but does not fully meet PLOS ONE’s publication criteria as it currently stands. Therefore, we invite you to submit a revised version of the manuscript that addresses this issue as described below.*

*Specifically, it is not clear how "efforts" is defined for making decisions and what does "enough efforts" mean.*

*It seems that more efforts is needed for difficult decisions, which should result in longer RT and larger or at least similar CIPC (if the efforts are not being wasted in distinguishing between the two options), which predict no relationship between RT and CIPC, instead of negative correlation as suggested in the manuscript.*

*Alternatively, the same amount of effort could mean the same amount of time or even less time on difficult decisions (because the rate of effort spent should be higher for difficult decisions), which predicts that difficult trials should be faster and thus more confident, as predicted by the negative correlation between RT and confidence (e.g., Kiani et al 2014; De Martino et al, 2013). In summary, the use of "effort" to explain the observed negative correlation with RT and CIPC does not seem well justified. Therefore, we suggest that instead of using this observation as an evidence supporting their hypothesis, the authors should accept their finding on the negative correlation between RT and CIPC as a piece of evidence that might not support their hypothesis.*

The relationship between RT and CIPC is an important but difficult point, and we acknowledge that we may have not have discussed it in a satisfying manner during the last review round. We agree that there are two distinct issues here:

First, effort really unfolds along two dimensions, namely: intensity and duration. This is because effort scales with the cumulative engagement of decision-related cognitive resources. From this perspective, trial-by-trial variations in RT are a good proxy for trial-buy-trial variations in effort only if effort intensity is somehow constant over trials. In particular, effort intensity may increase with information processing rate. If this were the case, and if such an increase in effort intensity somehow overcompensated the decrease in effort duration, then short RT would signal high effort. This implies that the relationship between RT and effort (e.g., the expected sign of the correlation between the two) depends on effort intensity, which we don’t have access to.

Second, even if RT was a reliable empirical proxy for effort, the expected relationship between RT and CIPC (under our hypothesis) would still be ambiguous. This is because it depends upon how decisions are triggered. We maintain that, under fairly consensual assumptions, one should predict that CIPC decreases when RT increases. As we quickly exemplify below, this happens under the assumptions of DDMs with collapsing bounds. This is, however, just one possibility and other alternative computational scenarios (that suggest different mechanisms for how decisions are triggered) may make qualitatively different predictions.

Taken together, this means that the negative correlation we see between CIPC and RT corroborates our working assumption under two additional tenets: (i) RT is a good proxy for effort, and (ii) decisions are triggered by a mechanism similar to a DDM with collapsing bounds. However, we acknowledge that our data cannot support these tenets. Therefore, this correlation may have other interpretations that do not relate to our working assumption. In other words, this correlation cannot be taken as evidence in favor of our working assumption. We have modified the corresponding Discussion paragraph accordingly (NB: for the sake of consistency with previous review rounds, we refrain from mentioning the notion of "effort" in this context):

In our experiment, the correlation between CIPC and RT turns out to be significant and negative, even when controlling for pre-choice decision variables (mean correlation = -0.1236, p<0.001). Can we interpret this result as providing evidence in favor or against our working assumption? Recall that we hypothesize that value reassessment during decision-making is a critical determinant of CIPC. This might tempt one to predict that longer RT should lead to greater CIPC. The logic of this idea would be that difficult decisions cause people to deliberate longer, which in turn allows for larger changes in value estimates. But in fact, predictions regarding the relationship between RT and CIPC actually depend upon two types of implicit assumptions. First, decision deliberation is likely to unfold along two dimensions, namely: intensity and duration. From this perspective, trial-by-trial variations in RT are a good proxy for trial-by-trial variations in decision deliberation only if intensity is constant over trials. But this may not be the case. In particular, information processing rate may vary. If such variations in intensity overcompensate for variations in duration, then short RT would signal intense decision deliberation. Second, even if RT were a reliable empirical proxy for decision deliberation, the expected relationship between RT and CIPC would remain ambiguous. This is because it would still depend upon how decisions are triggered. Consider, for example, drift-diffusion decision models of value-based decision making (Ratcliff et al., 2016; Tajima et al., 2016). Here, a decision is triggered when some decision variable (e.g., value difference) eventually reaches a collapsing bound. Importantly, those decisions that encounter little or no value spreading take more time because the system has to wait until the bound (i.e., the evidence that is demanded for triggering a decision) is small enough. In turn, choices with long RT would be those made with low confidence and small CIPC. Although the observed correlation between CIPC and RT corroborates this scenario, other alternative computational scenarios (that suggest different mechanisms for how decisions are triggered) may make qualitatively different predictions. Taken together, these issues suggest that the observed correlation between CIPC and RT cannot be taken as clear direct evidence that CIPC is driven by value reassessment that happens during the decision.

**A note on DDM with collapsing bounds**

In brief, optimal DDMs of value-based decision making state that (i) decisions are triggered when the decision variable (essentially the difference between option values) hits a predefined bound, (ii) and bounds are collapsing with time (Tajima et al., 2016). Figure 1 below shows two typical sample paths (i.e. two trials) simulated under the DDM with collapsing bounds.

Figure 1: Two exemplar simulations of the DDM with collapsing bounds. The blue and green traces show the decision variable stochastic dynamics (y-axis) as it evolves over time (x-axis). The collapsing bounds are shown in red.

One can see that those trials that effectively take longer to reach the collapsing bound (here: blue trace) correspond to situations in which the DV did (by chance) stay within a limited domain. In contrast, when the DV increases in magnitude (here: green trace), then the bound is hit sooner. This is a direct consequence of the collapsing bounds: the amount of evidence that is required for triggering a choice decreases as elapsed time increases. In other terms, the system triggers the decision despite weak evidence when elapsed time is long enough. As we will be clearer below, this simple phenomenon predicts that CIPC should decrease with RT.

Now Figure 2 below summarizes the results of 200 Monte-Carlo DDM simulations of the same DDM. In these simulations, the drift rate varies randomly at each trial, as well as the initial DV, and we collect the ensuing response times (RT) and final decision variables (DV) when the bound is hit. For the sake of completeness, the simulation code is appended below.

|  |  |  |
| --- | --- | --- |
| Figure 1: Monte-Carlo simulations of the DDM with collapsing bounds. Left: RT (y-axis) is plotted against drift rate (x-axis). Middle: final DV (y-axis) is plotted against drift rate (x-axis). Right: final DV (y-axis) is plotted against RT (x-axis). Each dot is a simulated trial (here, N=1000). Note that final DV can be negative, which means that the lower bound was hit (despite a positive drift rate). | | |

One can see that, when drift rate increases, RT decreases (left panel) and the final DV increases (middle panel). Note that -the absolute value of- drift rate is a proxy for choice ease, i.e. inverse choice difficulty. In addition, choice confidence increases with the -the absolute value of- DV (cf. our Results section). This means that under the DDM, when choice difficulty increases, RT increases and choice confidence decreases. In addition, DV decreases when RT increases (right panel), which implies that choice confidence should decrease when RT increases. As you highlighted, all these effects are evidenced from behavioral data in De Martino et al. (2013).

Now, under this scenario, the final DV can also be related to CIPC, because it can be seen as the accumulated difference between option values. More precisely, one may define CIPC as follows. Recall that the DDM predicts that the choice is determined by which bound is hit. Therefore, V_chosen_ - V_unchosen_ = sign(DV)*DV. And one can measure CIPC as the spreading of alternatives: SoA = sign(DV)*(DV-DV0), where DV is, by abuse of notation, the final DV, and DV0 is the initial DV. The ensuing relationships between CIPC on one hand, and either RT or final DV on the other hand are shown on Figure 3 below:

|  |  |
| --- | --- |
| Figure 2: Possible DDM predictions on CIPC. Left: CIPC (y-axis) is plotted against RT (x-axis). Left: CIPC (y-axis) is plotted against final DV (x-axis). | |

One can see that, under this scenario, CIPC decreases when RT increases, while it increases when choice confidence (|DV|) increases. Putting this scenario together with our working assumption goes as follows. Increasing choice difficulty does make people deliberate for longer. However, those trials that take the longest are those trials that did not yield much change in the DV (i.e. where the DV stayed within a limited range). In other words, those trials that encounter little or no value spreading take more time because the system has to wait until the bound (i.e. the evidence that is required for triggering a decision) is small enough. In brief, under the DDM with collapsing boundaries, one would expect CIPC to decrease when RT increases.

The matlab code we used to derive these simulations is appended below:

```
b = .01; % collapsing bound's decay rate
A = 1e2; % collapsing bound's magnitude
s = 1; % std[DDM's noise term]
Nmc = 2e2; % number of Monte-Carlo simulations
DR  = NaN(Nmc,1); % drift rate
DV = NaN(Nmc,1); % final decision variable (at bound hit)
DV0 = NaN(Nmc,1); % initial decision variable
RT  = NaN(Nmc,1); % response time
for imc=1:Nmc % loop over MC simulations
    % sample drift rate at random
    DR(imc) = abs(0.1*randn); % so that correct choices hit the upper bound
    % sample DDM initial condition
    DV0(imc) = randn; % small pre-choice value difference...
    % simulate DDM with collapsing bounds
    stop = 0;
    t = 1;
    dv = DV0(imc);
    dr = DR(imc);
    while ~stop % loop until the bound is hit
        dv = dv + dr + s*randn; % update DV
        dvt = A*exp(-b*t); % update bound
        if abs(dv)>=dvt % stopping rule
            stop = 1;
        else
            t = t + 1; % update time
        end
    end
    % store decision variables variables
    DV(imc) = dv; % final DV
    RT(imc) = t; % response time (bound hit)
end
CIPC = sign(DV).*(DV-DV0); % Spreading of Alternatives (SoA)

hf = figure;
ha = subplot(2,2,1,'parent',hf);
hp = plot(ha,DR,RT,'.');
xlabel(ha,'drift rate')
ylabel(ha,'RT')
ha = subplot(2,2,2,'parent',hf);
hp = plot(ha,DR,DV,'.');
xlabel(ha,'drift rate')
ylabel(ha,'DV')
ha = subplot(2,2,3,'parent',hf);
hp = plot(ha,RT,DV,'.');
xlabel(ha,'RT')
ylabel(ha,'DV')
hf2 = figure;
ha2 = subplot(2,2,1,'parent',hf2);
hp = plot(ha2,RT,CIPC,'.');
ylabel(ha2,'CIPC')
xlabel(ha2,'RT');
ha = subplot(2,2,2,'parent',hf2);
plot(ha,DV,CIPC,'.')
ylabel(ha,'CIPC')
xlabel(ha,'DV');
```
